# Poly[piperazine-1,4-diium [μ_4_-chlorido-μ_3_-chlorido-tri-μ_2_-chlorido-chloridodicadmate(II)] monohydrate]

**DOI:** 10.1107/S1600536812001626

**Published:** 2012-01-21

**Authors:** Marwa Adib, Meher El Glaoui, Pedro Sidonio Pereira da Silva, Manuela Ramos Silva, Cherif Ben Nasr

**Affiliations:** a Laboratoire de Chimie des Matériaux, Faculté des Sciences de Bizerte, 7021 Zarzouna, Tunisia; b CEMDRX, Physics Department, University of Coimbra, P-3004-516 Coimbra, Portugal

## Abstract

In the title compound, {(C_5_H_14_N_2_)[Cd_2_Cl_6_]·H_2_O}_*n*_, the asymmetric unit contains one piperazinediium cation, one [Cd_2_Cl_6_]^2−^ anion and a water mol­ecule. The coordination geometries of the two Cd^2+^ cations are distorted octa­hedral. Adjacent Cd^II^ atoms are inter­connected alternately by paired chloride bridges, generating polymeric chains parallel to [010]. Neighbouring chains are connected by O—H⋯Cl hydrogen bonds involving the water mol­ecules, forming layers at *z* = *n*/2. The crystal packing is further stabilized by inter­molecular N—H⋯Cl and N—H⋯O hydrogen bonds, one of which is bifurcated.

## Related literature

For general background to polymeric chlorido­cadmate(II) materials, see: Corradi *et al.* (1997 ▶). For the geometry around the Cd^II^ ion, see: Corradi *et al.* (1997 ▶, 1998 ▶); Xia *et al.* (2005 ▶); Jian *et al.* (2006 ▶); Partin & O Keeffe (1991 ▶). For Cd—Cl bond lengths, see: El Glaoui *et al.* (2010 ▶). For geometrical features of the organic cation, see: Yin & Wu (2010 ▶).
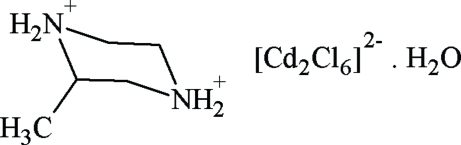


## Experimental

### 

#### Crystal data

(C_5_H_14_N_2_)[Cd_2_Cl_6_]·H_2_O
*M* *_r_* = 557.70
Monoclinic, 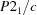

*a* = 12.1907 (3) Å
*b* = 6.8088 (2) Å
*c* = 21.4590 (5) Å
β = 120.521 (1)°
*V* = 1534.39 (7) Å^3^
*Z* = 4
Mo *K*α radiation
μ = 3.80 mm^−1^
*T* = 293 K
0.40 × 0.27 × 0.16 mm

#### Data collection

Bruker APEXII CCD area-detector diffractometer
Absorption correction: multi-scan (*SADABS*; Sheldrick, 2003 ▶) *T*_min_ = 0.411, *T*_max_ = 0.545
22206 measured reflections
3688 independent reflections
3449 reflections with *I* > 2σ(*I*)
*R*_int_ = 0.031

#### Refinement

*R*[*F* ^2^ > 2σ(*F* ^2^)] = 0.018
*wR*(*F* ^2^) = 0.045
*S* = 1.12
3688 reflections
154 parameters
H atoms treated by a mixture of independent and constrained refinement
Δρ_max_ = 0.48 e Å^−3^
Δρ_min_ = −0.73 e Å^−3^

### 

Data collection: *APEX2* (Bruker, 2003 ▶); cell refinement: *SAINT* (Bruker, 2003 ▶); data reduction: *SAINT*; program(s) used to solve structure: *SHELXS97* (Sheldrick, 2008 ▶); program(s) used to refine structure: *SHELXL97* (Sheldrick, 2008 ▶); molecular graphics: *SHELXTL* (Sheldrick, 2008 ▶); software used to prepare material for publication: *SHELXL97*.

## Supplementary Material

Crystal structure: contains datablock(s) global, I. DOI: 10.1107/S1600536812001626/lr2045sup1.cif

Structure factors: contains datablock(s) I. DOI: 10.1107/S1600536812001626/lr2045Isup2.hkl

Additional supplementary materials:  crystallographic information; 3D view; checkCIF report

## Figures and Tables

**Table 1:** Hydrogen-bond geometry (Å, °)

*D*—H⋯*A*	*D*—H	H⋯*A*	*D*⋯*A*	*D*—H⋯*A*
N1—H1*A*⋯Cl3^i^	0.90	2.32	3.0819 (17)	143
N1—H1*B*⋯Cl2	0.90	2.35	3.2451 (16)	171
N4—H4*A*⋯Cl6^ii^	0.90	2.44	3.1614 (17)	138
N4—H4*A*⋯O1*W*^iii^	0.90	2.45	3.131 (2)	133
N4—H4*B*⋯O1*W*^iv^	0.90	1.90	2.791 (2)	171
O1*W*—H1*W*⋯Cl6^v^	0.83 (4)	2.40 (4)	3.2140 (19)	166 (3)
O1*W*—H2*W*⋯Cl1^vi^	0.84 (4)	2.68 (4)	3.503 (2)	168 (3)

Symmetry codes: (i) 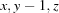; (ii) 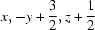; (iii) 
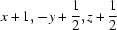
; (iv) 
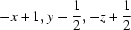
; (v) 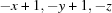; (vi) 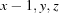.

**Fig. 1.**
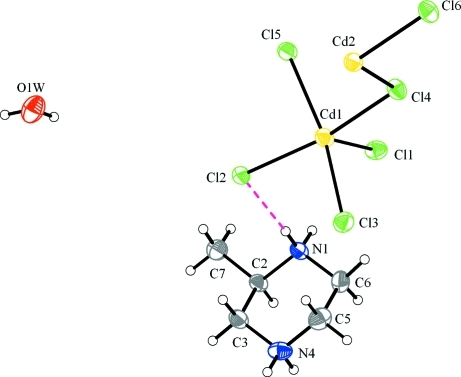
A view of (I) showing 50% probability displacement ellipsoids.

**Fig. 2.**
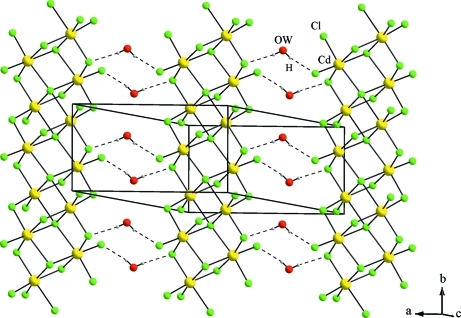
A view of the crystal structure of (I) showing the layer organization between Cd2Cl62- anion and H2O molecules. Dotted lines show intermolecular hydrogen bonding.

**Fig. 3.**
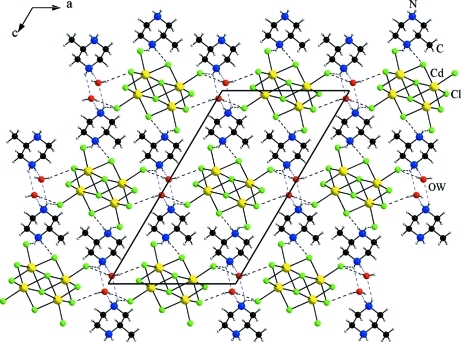
Packing diagram of the compound viewed down the b axis. Hydrogen bonds are shown as dashed lines.

## supplementary crystallographic information

### Comment

Polymeric chlorocadmates(II) represent a class of materials with chlorine atoms as ligands, connecting neighboring cadmium atoms, thus forming one or two- dimensional arrangements. One-dimensionality of metal chains allows easier modeling of physical properties and structure property correlations (Corradi *et al.*, 1997). In these compounds, the cadmium(II) cations exhibits a variety of coordination geometries and coordination numbers. However, it is worth to note that octahedral coordination of Cd^II^ is essentially present only in polymeric chlorocadmates(II), although a variety of stoichiometries are possible. The CdCl_6_ octahedra can form chains by face, edge, or vertex sharing [Corradi *et al.*, 1997; Xia *et al.*, 2005; Corradi *et al.*, 1998; Jian *et al.*, 2006). As a contribution to the investigation of the above materials, we report here the crystal structure of one such compound, Cd_2_Cl_6_C_5_H_14_N_2_H_2_O (I), formed from the reaction of piperazine, hydrochloride acid and cadmium chloride. The asymmetric unit of the title material contains one piperazinedium cation, one Cd_2_Cl_6_^2-^ anion and a water molecule (Fig. 1). Within Cd_2_Cl_6_ moiety, each Cd^II^ cations is coordinated by six chlorine atoms forming a distorted octahedron. Packing of Cd_2_Cl_6_C_5_H_14_N_2_H_2_O (Fig.2) shows that adjacent Cd ions are interconnected alternatively by paired chloride bridges to generate an infinite one-dimensional coordination chain crystallographic *b* axis. The closest Cd—Cd distance within the chain is 3.979 (1) A° is fairly close to the one determined in the one-dimensional chain of slightly distorted edge-charing octahedral(Partin & O Keeffe, 1991). These chains, situated at (1/2, 0, 0) and (1/2, 0, 1/2), are interconnected by the water molecules *via* O—H···Cl hydrogen bonds to form layers extending along the (a, c) plane at *z* = n/2 (Fig. 3). In the Cd_2_Cl_6_ entities, the Cd—Cl distances in the octahedra range between 2.4852 (5) and 2.9415 (5) A°. Cd—Cl distances of edge sharing chlorine atoms are 2.5449 (5) (Cd1—Cl4), 2.7148 (4) (Cd1—Cl5^i^), 2.6007 (5) (Cd2—Cl4) and 2.7293 (4) (Cd2—Cl5^i^) A° (symmetry codes in Table 1). The Cd—Cl—Cd bridges can thus be regarded as dissymmetric. These distances are longer than the terminal Cd—Cl ones 2.4852 (5) (Cd1—Cl3) and 2.51989 (5) (Cd2—Cl6) A°, which is typical of six coordinated Cd^II^ (El Glaoui *et al.*, 2010).. The Cl—Cd—Cl bond angles average close to 90.0° and range between 81.82 (1)° (for Cl2—Cd1—Cl5) and Cl3—Cd1—Cl4 97.18 (2)°, again confirming the close to octahedral nature of the CdCl_6_ building units. Overwise, owing to the obvious differences of Cd—Cl distances and Cl—Cd—Cl angles in Cd_2_Cl_6_C_5_H_14_N_2_H_2_O, the coordination geometry around the Cd atoms could be regarded as slightly distorted octahedron. The piperazinedium cations are anchored onto successive layers through N—H···Cl and N—H···O hydrogen bonds. The piperazinedium ring adopts a typical chair conformation and all the geometrical features agree with those found in the salt containing the same cation, 2-methylpiperazinediium tetrachlorozincate(II) (Yin & Wu, 2010). In this structure, the anionic and organic entities and the water molecules are connected through intricate O—H···Cl, N—H···Cl and N—H···O hydrogen bonding interactions, with one of these being three-center interactions, *viz*. N4—H4A···(Cl6^iv^, O1W^v^) (Fig. 3, details and symmetry codes in Table 1). It is worth noticing that the chlorine atoms Cl4 and Cl5 of the Cd_2_Cl_6_ are not involved in hydrogen bonding, while all hydrogen atoms that are attached to N1 and N4 nitrogen atoms are involved in hydrogen bondings.

### Experimental

A mixture of an aqueous solution of 2-methylpiperazine (3 mmol, 0.300 g), cadmium chloride (1.5 mmol, 0.275 g) and HCl (10 ml, 0.3 *M*) in a Petri dish was slowly evaporated at room temperature. Colorless single crystals of the title compound were isolated after several days (yield 63%).

### Refinement

All H atoms were located in a difference Fourier synthesis, placed in calculated positions and refined as riding on their parent atoms, using *SHELXL97* (Sheldrick, 2008) defaults.

### Crystal data

**Table:**

(C_5_H_14_N_2_)[Cd_2_Cl_6_]·H_2_O	*F*(000) = 1064
*M**_r_* = 557.70	*D*_x_ = 2.414 Mg m^−^^3^
Monoclinic, *P*2_1_/*c*	Mo *K*α radiation, λ = 0.71073 Å
Hall symbol: -P 2ybc	Cell parameters from 8164 reflections
*a* = 12.1907 (3) Å	θ = 2.7–28.0°
*b* = 6.8088 (2) Å	µ = 3.80 mm^−^^1^
*c* = 21.4590 (5) Å	*T* = 293 K
β = 120.521 (1)°	Block, colourless
*V* = 1534.39 (7) Å^3^	0.40 × 0.27 × 0.16 mm
*Z* = 4	

### Data collection

**Table:**

Bruker APEXII CCD area-detector diffractometer	3688 independent reflections
Radiation source: fine-focus sealed tube	3449 reflections with *I* > 2σ(*I*)
graphite	*R*_int_ = 0.031
φ and ω scans	θ_max_ = 28.0°, θ_min_ = 1.9°
Absorption correction: multi-scan (*SADABS*; Sheldrick, 2003)	*h* = −16→15
*T*_min_ = 0.411, *T*_max_ = 0.545	*k* = −8→8
22206 measured reflections	*l* = −28→26

### Refinement

**Table:**

Refinement on *F*^2^	Primary atom site location: structure-invariant direct methods
Least-squares matrix: full	Secondary atom site location: difference Fourier map
*R*[*F*^2^ > 2σ(*F*^2^)] = 0.018	Hydrogen site location: inferred from neighbouring sites
*wR*(*F*^2^) = 0.045	H atoms treated by a mixture of independent and constrained refinement
*S* = 1.12	*w* = 1/[σ^2^(*F*_o_^2^) + (0.0198*P*)^2^ + 0.7665*P*] where *P* = (*F*_o_^2^ + 2*F*_c_^2^)/3
3688 reflections	(Δ/σ)_max_ = 0.001
154 parameters	Δρ_max_ = 0.48 e Å^−^^3^
0 restraints	Δρ_min_ = −0.73 e Å^−^^3^

### Special details

**Table:**

Geometry. All e.s.d.'s (except the e.s.d. in the dihedral angle between two l.s. planes) are estimated using the full covariance matrix. The cell e.s.d.'s are taken into account individually in the estimation of e.s.d.'s in distances, angles and torsion angles; correlations between e.s.d.'s in cell parameters are only used when they are defined by crystal symmetry. An approximate (isotropic) treatment of cell e.s.d.'s is used for estimating e.s.d.'s involving l.s. planes.
Refinement. Refinement of *F*^2^ against ALL reflections. The weighted *R*-factor *wR* and goodness of fit *S* are based on *F*^2^, conventional *R*-factors *R* are based on *F*, with *F* set to zero for negative *F*^2^. The threshold expression of *F*^2^ > σ(*F*^2^) is used only for calculating *R*-factors(gt) *etc*. and is not relevant to the choice of reflections for refinement. *R*-factors based on *F*^2^ are statistically about twice as large as those based on *F*, and *R*- factors based on ALL data will be even larger.

### Fractional atomic coordinates and isotropic or equivalent isotropic displacement parameters (Å^2^)

**Table:**

	*x*	*y*	*z*	*U*_iso_*/*U*_eq_	
Cd1	0.676903 (13)	0.40204 (2)	0.082720 (7)	0.02594 (5)	
Cd2	0.646100 (13)	0.89968 (2)	−0.019721 (7)	0.02546 (5)	
Cl1	0.80264 (5)	0.07606 (7)	0.09737 (2)	0.02919 (10)	
Cl2	0.54286 (4)	0.25219 (7)	0.13506 (2)	0.02547 (9)	
Cl3	0.82700 (4)	0.53623 (8)	0.20479 (3)	0.03199 (10)	
Cl4	0.77287 (5)	0.57158 (7)	0.01573 (3)	0.02863 (10)	
Cl5	0.48547 (4)	0.24642 (7)	−0.04173 (2)	0.02682 (10)	
Cl6	0.74505 (5)	1.02346 (7)	−0.09068 (3)	0.03096 (10)	
N1	0.76370 (15)	−0.0550 (2)	0.23986 (8)	0.0255 (3)	
H1A	0.7654	−0.1453	0.2097	0.031*	
H1B	0.7095	0.0407	0.2127	0.031*	
N4	0.85051 (16)	0.0786 (2)	0.38410 (8)	0.0302 (4)	
H4A	0.8507	0.1670	0.4153	0.036*	
H4B	0.9034	−0.0199	0.4100	0.036*	
C2	0.71565 (17)	−0.1493 (3)	0.28474 (9)	0.0236 (4)	
H2	0.7724	−0.2584	0.3119	0.028*	
C3	0.71971 (18)	0.0001 (3)	0.33791 (10)	0.0282 (4)	
H3A	0.6620	0.1072	0.3117	0.034*	
H3B	0.6914	−0.0607	0.3682	0.034*	
C5	0.8968 (2)	0.1733 (3)	0.33924 (11)	0.0343 (4)	
H5A	0.9834	0.2194	0.3702	0.041*	
H5B	0.8438	0.2859	0.3143	0.041*	
C6	0.89316 (18)	0.0298 (3)	0.28479 (11)	0.0320 (4)	
H6A	0.9178	0.0961	0.2538	0.038*	
H6B	0.9537	−0.0750	0.3099	0.038*	
C7	0.58312 (19)	−0.2299 (3)	0.23657 (11)	0.0341 (4)	
H7A	0.5273	−0.1255	0.2079	0.051*	
H7B	0.5525	−0.2864	0.2659	0.051*	
H7C	0.5853	−0.3288	0.2053	0.051*	
O1W	0.00855 (17)	0.2486 (3)	0.04060 (10)	0.0431 (4)	
H1W	0.063 (4)	0.162 (6)	0.0509 (19)	0.090 (13)*	
H2W	−0.046 (3)	0.197 (5)	0.0481 (18)	0.080 (11)*	

### Atomic displacement parameters (Å^2^)

**Table:**

	*U*^11^	*U*^22^	*U*^33^	*U*^12^	*U*^13^	*U*^23^
Cd1	0.02794 (8)	0.02668 (8)	0.02527 (8)	−0.00503 (5)	0.01503 (6)	−0.00082 (5)
Cd2	0.02605 (8)	0.02669 (8)	0.02454 (8)	−0.00547 (5)	0.01350 (6)	−0.00232 (5)
Cl1	0.0319 (2)	0.0269 (2)	0.0251 (2)	0.00093 (18)	0.01181 (19)	−0.00354 (17)
Cl2	0.0243 (2)	0.0304 (2)	0.02289 (19)	−0.00335 (17)	0.01289 (17)	0.00013 (17)
Cl3	0.0277 (2)	0.0360 (3)	0.0297 (2)	0.00006 (19)	0.01262 (19)	−0.0073 (2)
Cl4	0.0334 (2)	0.0273 (2)	0.0313 (2)	0.00249 (18)	0.0209 (2)	0.00483 (18)
Cl5	0.0293 (2)	0.0287 (2)	0.0237 (2)	−0.00274 (18)	0.01440 (18)	−0.00028 (17)
Cl6	0.0367 (2)	0.0281 (2)	0.0349 (2)	−0.00504 (19)	0.0231 (2)	0.00181 (19)
N1	0.0279 (8)	0.0309 (8)	0.0210 (7)	0.0022 (6)	0.0148 (6)	0.0015 (6)
N4	0.0374 (9)	0.0261 (9)	0.0221 (7)	0.0039 (7)	0.0115 (7)	−0.0021 (6)
C2	0.0262 (9)	0.0252 (9)	0.0212 (8)	0.0008 (7)	0.0135 (7)	0.0034 (7)
C3	0.0312 (10)	0.0327 (11)	0.0248 (9)	0.0028 (8)	0.0172 (8)	−0.0002 (8)
C5	0.0325 (10)	0.0302 (11)	0.0348 (10)	−0.0052 (9)	0.0131 (9)	0.0006 (9)
C6	0.0248 (9)	0.0397 (12)	0.0337 (10)	−0.0003 (8)	0.0164 (8)	0.0035 (9)
C7	0.0307 (10)	0.0410 (12)	0.0302 (10)	−0.0071 (9)	0.0151 (8)	−0.0030 (9)
O1W	0.0328 (9)	0.0374 (10)	0.0524 (10)	−0.0048 (7)	0.0167 (8)	−0.0072 (8)

### Geometric parameters (Å, °)

**Table:**

Cd1—Cl3	2.4852 (5)	N1—H1B	0.9000
Cd1—Cl4	2.5449 (5)	N4—C5	1.488 (3)
Cd1—Cl2	2.6147 (4)	N4—C3	1.485 (2)
Cd1—Cl1	2.6239 (5)	N4—H4A	0.9000
Cd1—Cl5	2.7148 (4)	N4—H4B	0.9000
Cd1—Cl5^i^	2.9415 (5)	C2—C7	1.511 (3)
Cd2—Cl6	2.5198 (5)	C2—C3	1.510 (3)
Cd2—Cl1^ii^	2.5548 (5)	C2—H2	0.9800
Cd2—Cl2^i^	2.5905 (4)	C3—H3A	0.9700
Cd2—Cl4	2.6007 (5)	C3—H3B	0.9700
Cd2—Cl5^i^	2.7293 (4)	C5—C6	1.506 (3)
Cd2—Cl5^ii^	2.9483 (5)	C5—H5A	0.9700
Cl1—Cd2^iii^	2.5549 (5)	C5—H5B	0.9700
Cl2—Cd2^i^	2.5904 (4)	C6—H6A	0.9700
Cl5—Cd2^i^	2.7293 (4)	C6—H6B	0.9700
Cl5—Cd1^i^	2.9415 (5)	C7—H7A	0.9600
Cl5—Cd2^iii^	2.9482 (5)	C7—H7B	0.9600
N1—C6	1.486 (2)	C7—H7C	0.9600
N1—C2	1.502 (2)	O1W—H1W	0.83 (4)
N1—H1A	0.9000	O1W—H2W	0.84 (4)
			
Cl3—Cd1—Cl4	97.183 (17)	C2—N1—H1A	109.1
Cl3—Cd1—Cl2	88.677 (16)	C6—N1—H1B	109.1
Cl4—Cd1—Cl2	170.588 (16)	C2—N1—H1B	109.1
Cl3—Cd1—Cl1	96.435 (16)	H1A—N1—H1B	107.8
Cl4—Cd1—Cl1	92.478 (16)	C5—N4—C3	110.85 (14)
Cl2—Cd1—Cl1	94.181 (16)	C5—N4—H4A	109.5
Cl3—Cd1—Cl5	170.147 (15)	C3—N4—H4A	109.5
Cl4—Cd1—Cl5	91.920 (15)	C5—N4—H4B	109.5
Cl2—Cd1—Cl5	81.819 (14)	C3—N4—H4B	109.5
Cl1—Cd1—Cl5	86.892 (14)	H4A—N4—H4B	108.1
Cl3—Cd1—Cl5^i^	92.263 (15)	N1—C2—C7	110.25 (14)
Cl4—Cd1—Cl5^i^	83.946 (14)	N1—C2—C3	108.81 (15)
Cl2—Cd1—Cl5^i^	88.486 (14)	C7—C2—C3	112.00 (16)
Cl1—Cd1—Cl5^i^	170.954 (14)	N1—C2—H2	108.6
Cl5—Cd1—Cl5^i^	84.932 (14)	C7—C2—H2	108.6
Cl6—Cd2—Cl1^ii^	95.130 (16)	C3—C2—H2	108.6
Cl6—Cd2—Cl2^i^	91.288 (15)	N4—C3—C2	111.02 (15)
Cl1^ii^—Cd2—Cl2^i^	170.043 (16)	N4—C3—H3A	109.4
Cl6—Cd2—Cl4	93.917 (16)	C2—C3—H3A	109.4
Cl1^ii^—Cd2—Cl4	94.589 (16)	N4—C3—H3B	109.4
Cl2^i^—Cd2—Cl4	92.555 (16)	C2—C3—H3B	109.4
Cl6—Cd2—Cl5^i^	173.207 (15)	H3A—C3—H3B	108.0
Cl1^ii^—Cd2—Cl5^i^	91.424 (15)	N4—C5—C6	110.43 (17)
Cl2^i^—Cd2—Cl5^i^	81.980 (14)	N4—C5—H5A	109.6
Cl4—Cd2—Cl5^i^	87.324 (14)	C6—C5—H5A	109.6
Cl6—Cd2—Cl5^ii^	96.792 (15)	N4—C5—H5B	109.6
Cl1^ii^—Cd2—Cl5^ii^	83.379 (14)	C6—C5—H5B	109.6
Cl2^i^—Cd2—Cl5^ii^	88.316 (14)	H5A—C5—H5B	108.1
Cl4—Cd2—Cl5^ii^	169.235 (14)	N1—C6—C5	111.02 (16)
Cl5^i^—Cd2—Cl5^ii^	82.170 (14)	N1—C6—H6A	109.4
Cd2^iii^—Cl1—Cd1	100.407 (17)	C5—C6—H6A	109.4
Cd2^i^—Cl2—Cd1	101.062 (15)	N1—C6—H6B	109.4
Cd1—Cl4—Cd2	100.341 (16)	C5—C6—H6B	109.4
Cd1—Cl5—Cd2^i^	95.136 (14)	H6A—C6—H6B	108.0
Cd1—Cl5—Cd1^i^	95.067 (14)	C2—C7—H7A	109.5
Cd2^i^—Cl5—Cd1^i^	88.267 (13)	C2—C7—H7B	109.5
Cd1—Cl5—Cd2^iii^	89.184 (13)	H7A—C7—H7B	109.5
Cd2^i^—Cl5—Cd2^iii^	97.829 (14)	C2—C7—H7C	109.5
Cd1^i^—Cl5—Cd2^iii^	172.243 (17)	H7A—C7—H7C	109.5
C6—N1—C2	112.43 (14)	H7B—C7—H7C	109.5
C6—N1—H1A	109.1	H1W—O1W—H2W	105 (3)
			
Cl3—Cd1—Cl1—Cd2^iii^	173.772 (17)	Cl1—Cd1—Cl5—Cd2^i^	95.163 (16)
Cl4—Cd1—Cl1—Cd2^iii^	−88.710 (18)	Cl5^i^—Cd1—Cl5—Cd2^i^	−88.711 (14)
Cl2—Cd1—Cl1—Cd2^iii^	84.634 (17)	Cl4—Cd1—Cl5—Cd1^i^	−83.748 (15)
Cl5—Cd1—Cl1—Cd2^iii^	3.079 (16)	Cl2—Cd1—Cl5—Cd1^i^	89.194 (14)
Cl3—Cd1—Cl2—Cd2^i^	176.878 (18)	Cl1—Cd1—Cl5—Cd1^i^	−176.127 (16)
Cl1—Cd1—Cl2—Cd2^i^	−86.765 (17)	Cl5^i^—Cd1—Cl5—Cd1^i^	0.0
Cl5—Cd1—Cl2—Cd2^i^	−0.517 (14)	Cl4—Cd1—Cl5—Cd2^iii^	89.754 (14)
Cl5^i^—Cd1—Cl2—Cd2^i^	84.579 (16)	Cl2—Cd1—Cl5—Cd2^iii^	−97.304 (14)
Cl3—Cd1—Cl4—Cd2	−88.832 (18)	Cl1—Cd1—Cl5—Cd2^iii^	−2.625 (14)
Cl1—Cd1—Cl4—Cd2	174.361 (16)	Cl5^i^—Cd1—Cl5—Cd2^iii^	173.501 (18)
Cl5—Cd1—Cl4—Cd2	87.391 (17)	C6—N1—C2—C7	179.04 (17)
Cl5^i^—Cd1—Cl4—Cd2	2.695 (15)	C6—N1—C2—C3	55.8 (2)
Cl6—Cd2—Cl4—Cd1	−176.201 (16)	C5—N4—C3—C2	59.2 (2)
Cl1^ii^—Cd2—Cl4—Cd1	88.323 (18)	N1—C2—C3—N4	−57.26 (19)
Cl2^i^—Cd2—Cl4—Cd1	−84.734 (17)	C7—C2—C3—N4	−179.41 (16)
Cl5^i^—Cd2—Cl4—Cd1	−2.891 (16)	C3—N4—C5—C6	−57.2 (2)
Cl5^ii^—Cd2—Cl4—Cd1	9.69 (9)	C2—N1—C6—C5	−55.5 (2)
Cl4—Cd1—Cl5—Cd2^i^	−172.459 (15)	N4—C5—C6—N1	55.1 (2)
Cl2—Cd1—Cl5—Cd2^i^	0.484 (13)		

Symmetry codes: (i) −*x*+1, −*y*+1, −*z*; (ii) *x*, *y*+1, *z*; (iii) *x*, *y*−1, *z*.

### Hydrogen-bond geometry (Å, °)

**Table:**

*D*—H···*A*	*D*—H	H···*A*	*D*···*A*	*D*—H···*A*
N1—H1A···Cl3^iii^	0.90	2.32	3.0819 (17)	143
N1—H1B···Cl2	0.90	2.35	3.2451 (16)	171
N4—H4A···Cl6^iv^	0.90	2.44	3.1614 (17)	138
N4—H4A···O1W^v^	0.90	2.45	3.131 (2)	133
N4—H4B···O1W^vi^	0.90	1.90	2.791 (2)	171
O1W—H1W···Cl6^i^	0.83 (4)	2.40 (4)	3.2140 (19)	166 (3)
O1W—H2W···Cl1^vii^	0.84 (4)	2.68 (4)	3.503 (2)	168 (3)

Symmetry codes: (iii) *x*, *y*−1, *z*; (iv) *x*, −*y*+3/2, *z*+1/2; (v) *x*+1, −*y*+1/2, *z*+1/2; (vi) −*x*+1, *y*−1/2, −*z*+1/2; (i) −*x*+1, −*y*+1, −*z*; (vii) *x*−1, *y*, *z*.
